# Skin wound closure delay in metabolic syndrome correlates with SCF deficiency in keratinocytes

**DOI:** 10.1038/s41598-020-78244-y

**Published:** 2020-12-10

**Authors:** Zhenping Wang, Yanhan Wang, Nicholas Bradbury, Carolina Gonzales Bravo, Bernd Schnabl, Anna Di Nardo

**Affiliations:** 1 Department of Dermatology, School of Medicine, University of California, San Diego, La Jolla, CA 92093 USA; 2 Division of Gastroenterology, School of Medicine, University of California, San Diego, La Jolla, CA 92093 USA

**Keywords:** Cell growth, Cell migration, Mechanisms of disease, Metabolic disorders, Skin diseases

## Abstract

Poor wound closure due to diabetes, aging, stress, obesity, alcoholism, and chronic disease affects millions of people worldwide. Reasons wounds will not close are still unclear, and current therapies are limited. Although stem cell factor (SCF), a cytokine, is known to be important for wound repair, the cellular and molecular mechanisms of SCF in wound closure remain poorly understood. Here, we found that SCF expression in the epidermis is decreased in mouse models of delayed wound closure intended to mimic old age, obesity, and alcoholism. By using SCF conditionally knocked out mice, we demonstrated that keratinocytes’ autocrine production of SCF activates a transient c-kit receptor in keratinocytes. Transient activation of the c-kit receptor induces the expression of growth factors and chemokines to promote wound re-epithelialization by increasing migration of skin cells (keratinocytes and fibroblasts) and immune cells (neutrophils) to the wound bed 24–48 h post-wounding. Our results demonstrate that keratinocyte-produced SCF is essential to wound closure due to the increased recruitment of a unique combination of skin cells and immune cells in the early phase after wounding. This discovery is imperative for developing clinical strategies that might improve the body’s natural repair mechanisms for treating patients with wound-closure pathologies.

## Introduction

After an injury occurs, wound repair requires activation and coordination of complex intracellular and intercellular pathways^1^. Although minor injuries in healthy people generally close well, larger wounds or the presence of various physiological or disease states (i.e. age, obesity, diabetes, and cancer) can impede the closure process in ways that are not well understood^2^. Delayed wound closure is a significant medical problem that reduces the quality of life and can cause an economic burden. For instance, the annual treatment cost of diabetic lower extremity ulcers alone exceeds 1.5 billion dollars^3^. An incomplete understanding of the cellular and molecular basis of wound repair has led to a lack of therapies for treating nonhealing wounds or for speeding up the repair of acute wounds and reducing scar formation^4^. Stem cell factor (SCF) is known to play an essential role in hematopoiesis, spermatogenesis, and melanogenesis^5,6^. Our prior work also revealed that epidermal keratinocytes maintain mast cells and melanocytes in the skin through the production of SCF^6^. SCF and its c-kit receptor are expressed by various skin cells, including keratinocytes, fibroblasts, mast cells, melanocytes, and endothelial cells^7–10^. The SCF-c-kit pathway was also demonstrated with subsequent auto- and paracrine activation of c-kit/JAK1/STAT3 signaling to induce growth factors, cell proliferation, migration, and tissue regeneration^11,12^. A few studies have shown that SCF is important for wound closure^13–15^; e.g. Zgheib et al. reported that SCF treatment accelerates wound closure in diabetic mice in part by increasing the recruitment of stem cells in the skin^14^. However, no studies have been conducted on the role of SCF production by keratinocytes in wound closure. In this study, we investigated keratinocyte-produced SCF in wound closure, production of growth factors and chemokines, and skin cells/immune cells early recruitment during the wound closure process by utilizing different mouse models including SCF conditionally knocked out K14cre Scf^fl/fl^ mice, aged mice, high-fat diet mouse model, and alcohol diet mouse model.

## Results

### Decreased SCF expression in the skin in different mouse models of delayed wound closure

**Figure 1:**
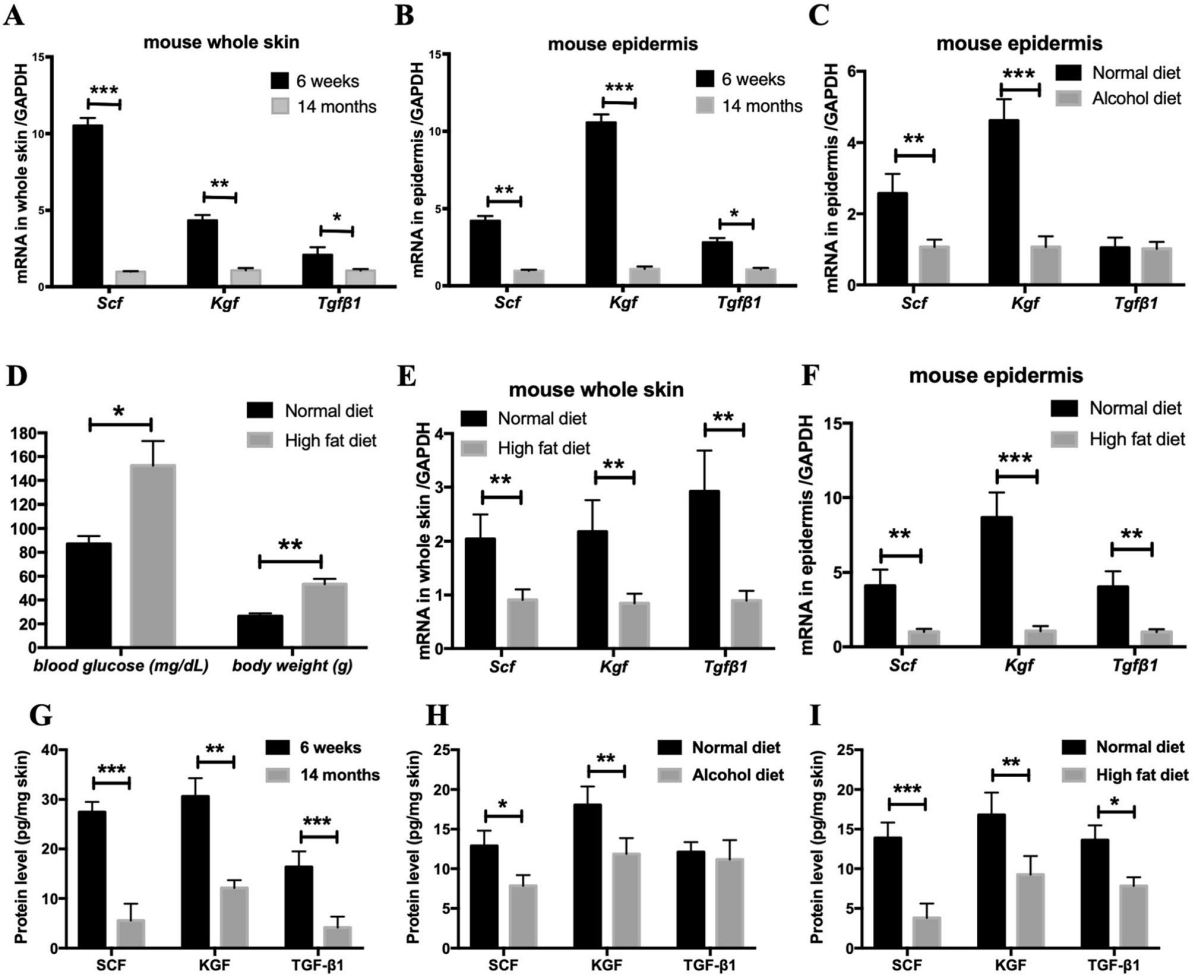
Decreased SCF expression in skin in different mouse models of delayed wound closure. (**A**,**B**) qPCR analysis for Scf, Kgf, and Tgf-β1 in whole skin and epidermis of aged mice and young mice; (**C**) qPCR analysis for Scf, Kgf, and Tgf-β1 in epidermis of chronic-binge ethanol diet mice; (**D**) Blood glucose and body weight of high-fat diet mice; (**E**,**F**) qPCR analysis for Scf, Kgf, and Tgf-β1 in whole skin and epidermis of high-fat diet mice; (**G**) SCF, KGF and TGF-β1 protein levels in whole skin of aged mice and young mice; (**H**) Protein levels of SCF, KGF and TGF-β1in whole skin of chronic-binge ethanol diet mice; (**I**) Protein levels of SCF, KGF and TGF-β1in whole skin of high-fat diet mice (*p < 0.05, **p < 0.01, ***p < 0.001).

Studies have shown that wound closure is markedly delayed in populations with aging, obesity, or alcoholism, a finding that is also supported by relevant murine models^16–18^. However, it is unknown whether the SCF levels in these models are affected. To address this gap, we assessed SCF expression in the skin of these confirmed murine models, including aged mice (14-month old mice), high-fat diet mice, and chronic-binge ethanol diet mice. As shown in Fig. 1, not only did SCF mRNA levels decrease, KGF (keratinocyte growth factor, also known as FGF7, a growth factor present in the epithelialization-phase of wound closure^19^) and TGF-β1 (a growth factor with critical roles in different phases of wound closure^20^) expressions were also significantly reduced in the whole skin of aged mice and high-fat diet mice (Fig. 1A,E). The high-fat diet mice also showed significantly increased blood glucose and body weight (Fig. 1D). Because keratinocytes are the primary source of growth factors, which may stimulate not only keratinocytes themselves but also fibroblasts or other mesenchymal cell types in the wound closure process^19^, we isolated the epidermis from the whole skin and found that SCF, KGF, and TGF-β1 mRNA levels decreased significantly in the epidermis of aged mice and high-fat diet mice (Fig. 1B,F). For the chronic-binge ethanol diet mouse model, we also found a significant decrease in SCF and KGF expressions in the epidermis (Fig. 1C). Given that keratinocytes makeup over 90% of the cells of the epidermis and thus constitute an important component of wound closure, our results suggest that SCF derived from keratinocytes plays a vital role in wound repair. Subsequent analysis of the protein levels of SCF, KGF, and TGF-β1 corroborated the qPCR gene expression data with highly similar trends; these results are shown in (Fig. 1G–I).

### SCF deficiency in keratinocytes delayed wound closure in skin

**Figure 2:**
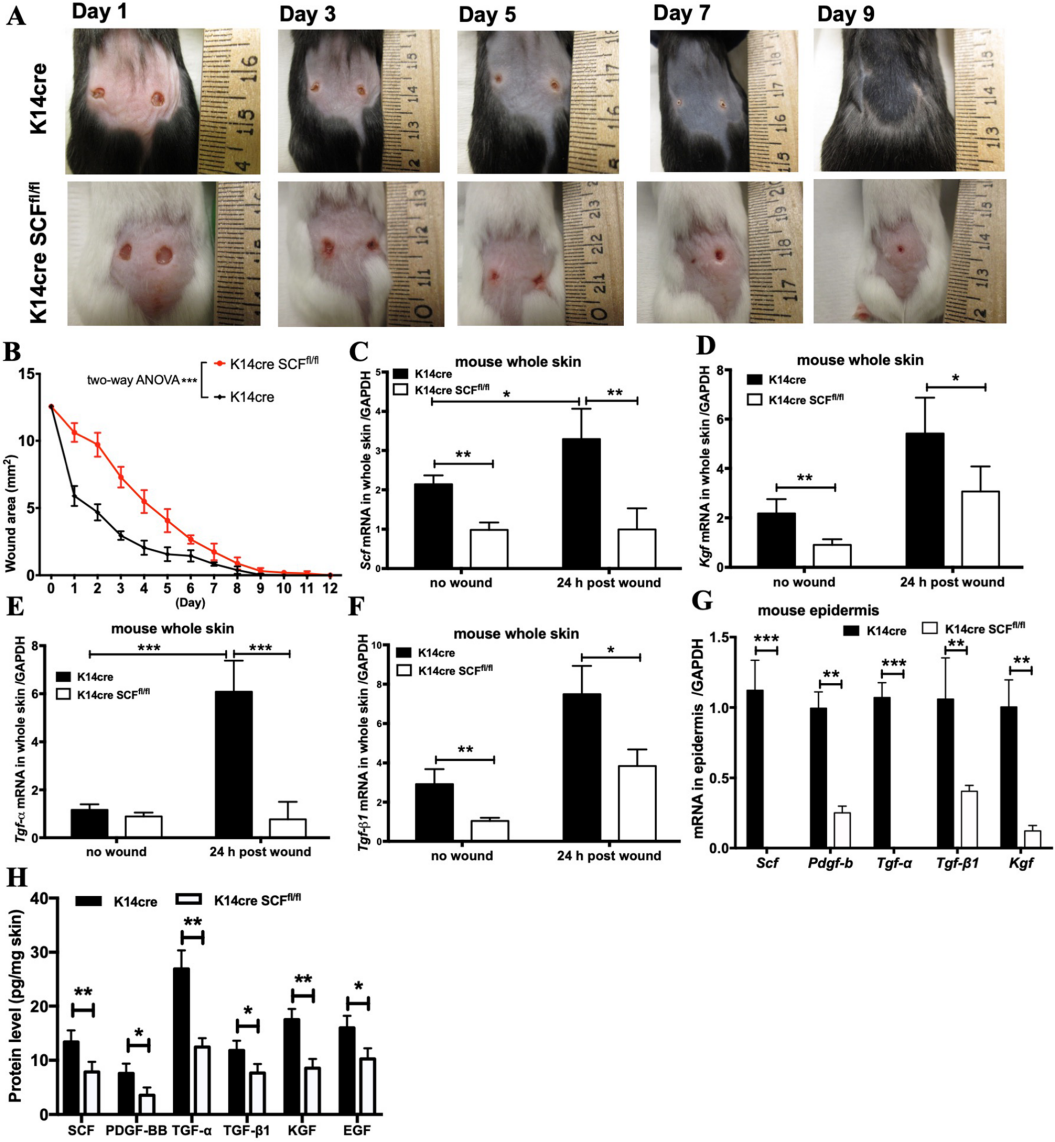
SCF deficiency in keratinocytes delayed wound closure in skin. (**A**,**B**) Representative wound area pictures and enumeration in mouse skin on different day; (**C**,**F**) qPCR analysis for Scf, Kgf, Tgf-α, and Tgf-β1 in whole skin of mice 24 h after wounding; (**G**) qPCR analysis for Scf, Pdgf-b, Kgf, Tgf-α, and Tgf-β1 in epidermis of mice; (**H**) SCF, PDGF-BB, KGF, TGF-α, TGF-β1, and EGF protein levels in whole skin of mice (*p < 0.05, **p < 0.01, ***p < 0.001).

To further determine the relative importance of keratinocyte-produced SCF in supporting wound closure, we bred K14cre transgenic mice that express Cre recombinase under the control of the keratin 14 promoter with mice containing floxed *Scf* alleles (Scf^fl/fl^ mice) to generate K14cre Scf^fl/fl^ mice, which lack SCF in a keratinocyte-specific manner. These mice are albino, viable, fertile, normal in size, and do not display any gross physical or behavioral abnormalities. Our data also confirmed that these mice have no SCF protein in epidermal keratinocytes, whereas their dermal fibroblasts can still express SCF^6^. To make a dorsal full-thickness skin wound, punch biopsies (4-mm) were used on the backs of the mice. To evaluate wound closure, the wound area was measured using the ImageJ software (National Institutes of Health) daily. Our results showed that the wounds of K14cre littermate controls were significantly smaller than that of K14cre Scf^fl/fl^ mice on days 1–6. On day 9, the wounds of K14cre control mice were completely closed, while the wounds of K14cre Scf^fl/fl^ mice were closed on day 12 (Fig. 2A,B). In addition, K14cre control mice expressed significantly more SCF, KGF, TGF-β1, and TGF-α (a growth factor predominantly expressed in keratinocytes that has a profound autocrine effect on keratinocytes in wound closure^19^) in the whole skin 24 h after wounding compared to K14cre Scf^fl/fl^ mice (Fig. 2C–F). To confirm these changes in keratinocytes, we isolated the epidermis from the whole skin. As shown in Fig. 2G, we found that when knocking out SCF from keratinocytes, the epidermis of K14cre Scf^fl/fl^ mice expressed significantly lower mRNA levels of KGF, TGF-β1, TGF-α and PDGF-b (platelet-derived growth factor subunit b, a predominantly keratinocyte-derived growth factor acting in a paracrine manner after wounding^19^). These results demonstrate that keratinocyte-derived SCF is critical for the production of main growth factors involved in wound closure and led to the hypothesis that keratinocytes might be key players in wound closure by producing SCF. To corroborate our findings in Fig. 2G, we analyzed the KGF, TGF-β1, TGF-α, PDGF-b, and EGF protein levels of whole mouse skin (Fig. 2H).

### SCF deficiency impaired the migration of keratinocytes

**Figure 3:**
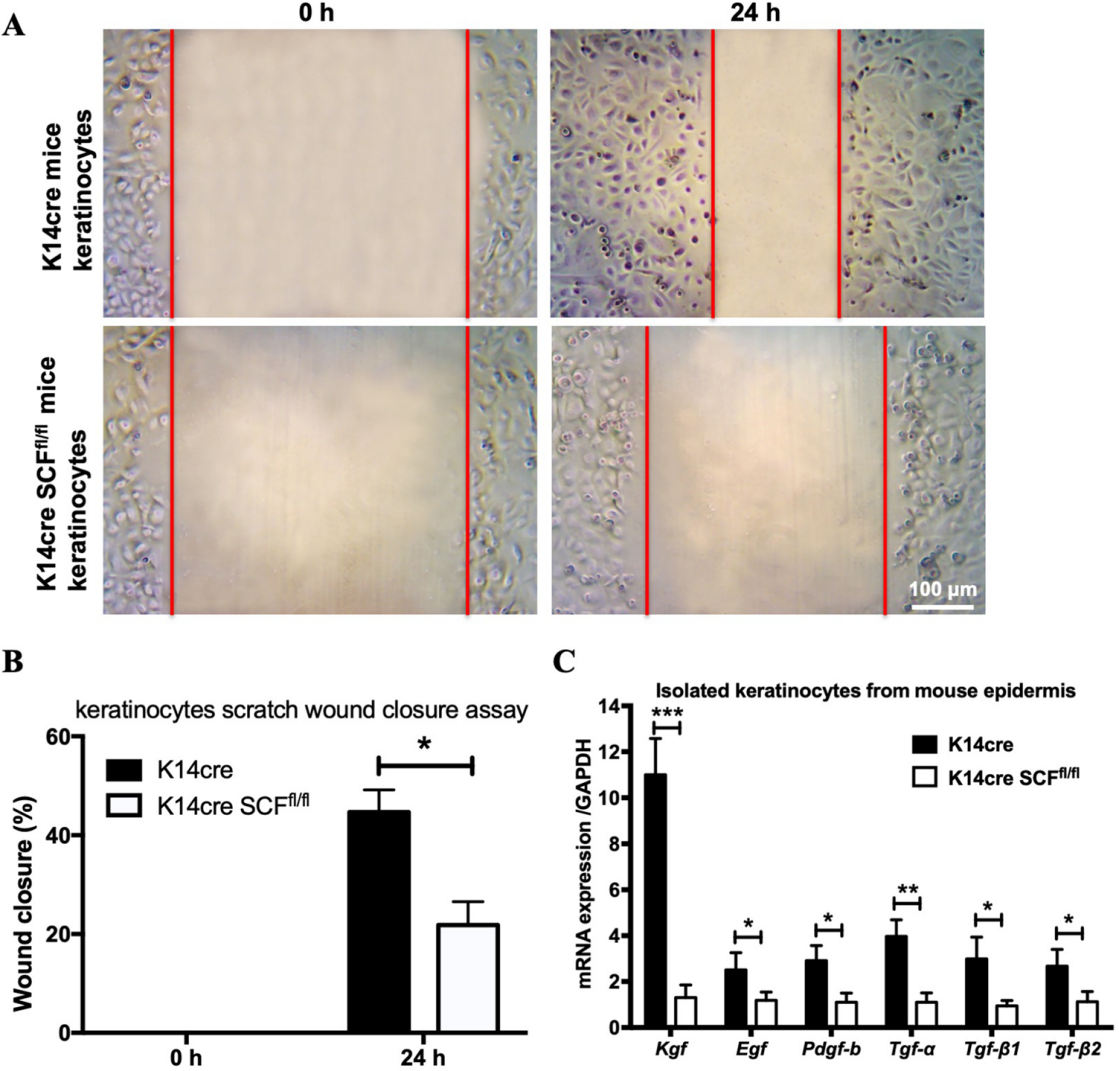
SCF deficiency impaired the migration of keratinocytes. (**A**,**B**) keratinocytes in vitro scratch wound closure representative pictures and enumeration; (**C**) qPCR analysis for Kgf, Pdgf-b, Egf, Tgf-α, Tgf-β1, and Tgf-β2 in isolated keratinocytes from epidermis of mice (*p < 0.05, **p < 0.01, ***p < 0.001).

Since delayed wound closure can be due to reduced keratinocyte migration or proliferation^19^, we isolated keratinocytes from mouse epidermis and performed an in vitro scratch wound closure assay to assess SCF’s the importance in keratinocyte migration. We found that K14cre control keratinocytes affected wound closure more rapidly than SCF-deficient K14cre SCF^fl/fl^ keratinocytes in a scratch assay. At 24 h when the extent of wound closure was 45% ± 4.5% for K14cre keratinocytes, the extent of closure for K14cre SCF^fl/fl^ keratinocytes scratch assay was only 22% ± 4.8% (Fig. 3A,B). Furthermore, complete wound closure of all scratches was seen in K14cre keratinocytes cultures by 72 h, compared with only half of the scratches for K14cre SCF^fl/fl^ keratinocytes. Therefore, K14cre keratinocytes migrated faster than the K14cre SCF^fl/fl^ keratinocytes to fill the scratch area, which correlated significantly with higher expressions of growth factors including KGF, EGF (epidermal growth factor), PDGF-b, TGF-α, TGF-β1 and TGF-β2 in K14cre keratinocytes (Fig. 3C). These results suggest a central pathogenetic role of keratinocyte-derived SCF in wound closure. More importantly, this illustrates that keratinocyte-derived SCF is necessary to induce keratinocyte migration, suggesting that keratinocytes possess c-kit receptors for SCF. These findings are consistent with previous evidence which showed that SCF and its c-kit receptor are co-expressed by epidermal keratinocytes^6,10,21^.

### SCF deficiency in keratinocytes impaired the recruitment of normal fibroblasts

**Figure 4:**
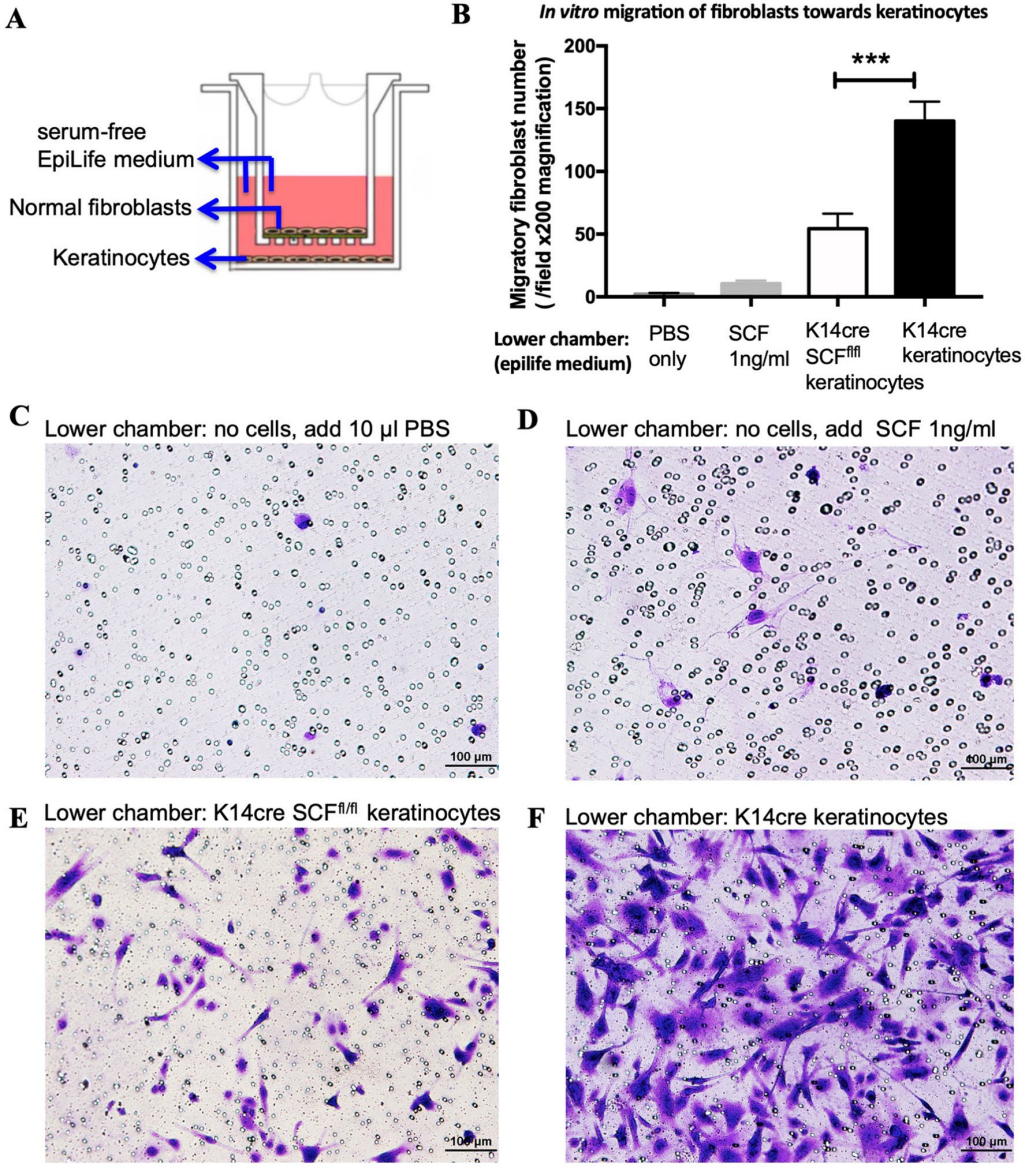
SCF deficiency in keratinocytes impaired the recruitment of normal fibroblasts. (**A**) Diagram of modified Boyden chamber transwell system; (**B**) The number of migratory fibroblasts after 24-h migration; (**C**–**F**) Representative pictures of migratory fibroblasts that had traversed the membrane stained with 0.1% crystal violet (***p < 0.001).

It is well known that keratinocytes and fibroblasts both play a crucial role in wound closure. Fibroblasts migrate into the wound bed, synthesize new extracellular matrix, and express thick actin bundles as myofibroblasts^22^. To determine whether SCF-deficient keratinocytes can undermine normal fibroblast’s migration towards keratinocytes, we performed a cell migration assay by using a modified Boyden chamber transwell with a membrane pore size of 8 μm (Fig. 4A). Lower and upper chambers were filled with serum-free EpiLife medium supplemented with 40 µM CaCl_2_ and EpiLife Defined Growth Supplement (Invitrogen) which supports keratinocytes growth and the formation of a cell monolayer in the lower chamber. This system can keep normal functioning fibroblasts in the upper chamber without proliferation for 24–72 h (fibroblasts proliferation data not shown). After 24-h migration, non-migratory fibroblasts were carefully removed by a cotton swab from the upper surface of the inserts. Migratory fibroblasts that had traversed the membrane were stained with 0.1% crystal violet (Sigma) and counted in three independent fields under microscopy. We found that there were less than ten migratory fibroblasts if a keratinocyte monolayer was absent in the lower chamber, suggesting that SCF alone is not the only reason for fibroblasts migration (Fig. 4B–D). When keratinocytes were present in the lower chamber, we observed typical migration of fibroblasts and found that K14cre keratinocytes induced significantly more migratory fibroblasts than SCF-deficient K14cre SCF^fl/fl^ keratinocytes (Fig. 4B,E,F). These results indicate that the ability of SCF-deficient keratinocytes to recruit normal fibroblasts is weakened because they express significantly less PDGF-b, TGF-α, TGF-β1, and TGF-β2 than K14cre control keratinocytes (Fig. 3C). Growth factors PDGF-b, TGF-α, TGF-β1, and TGF-β2 are also potent chemotactic factors for fibroblast recruitment in wound closure^19,23^.

### SCF deficiency in keratinocytes reduced early neutrophil recruitment after wounding

**Figure 5:**
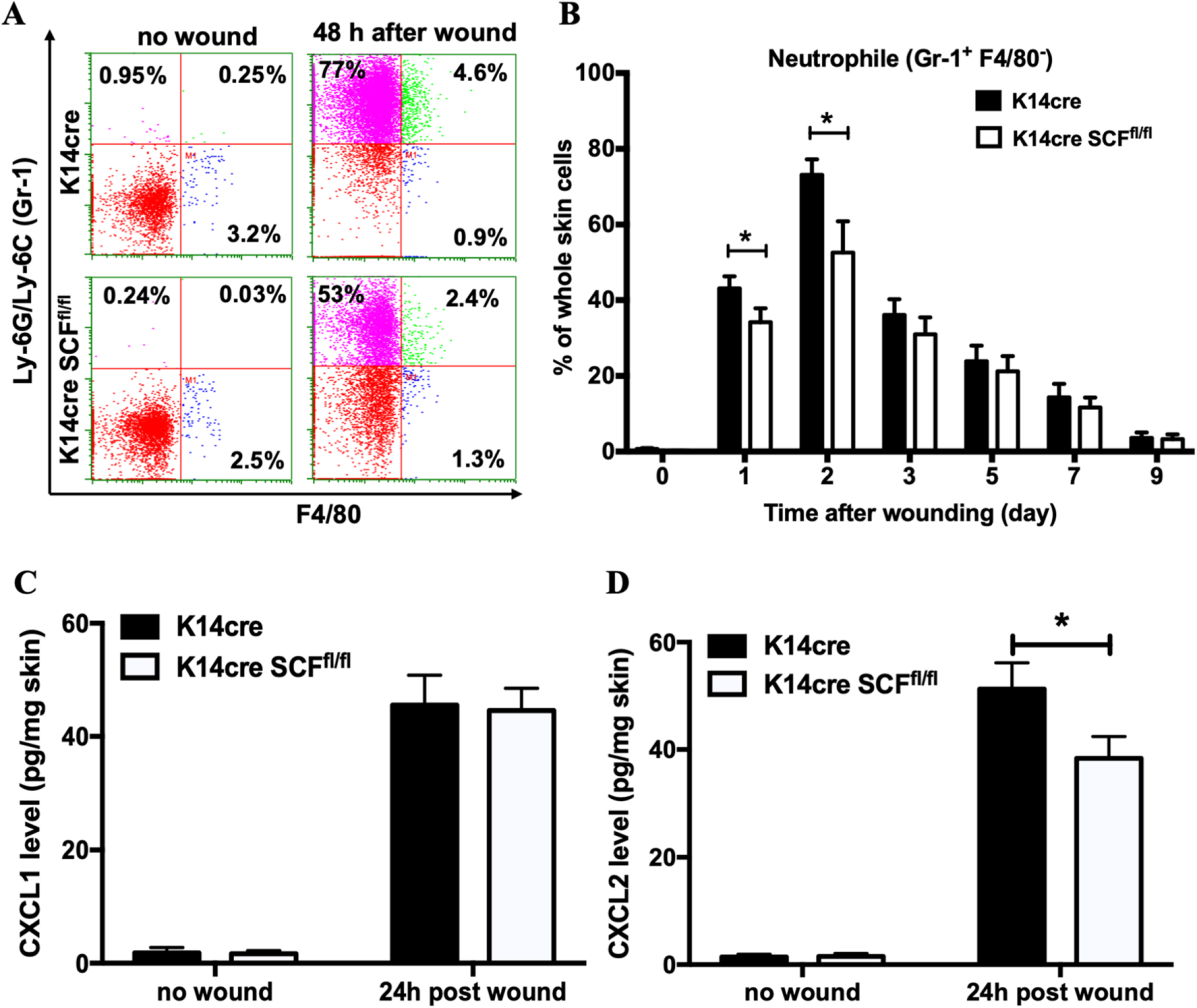
SCF deficiency in keratinocytes reduced early neutrophil recruitment after wounding. (**A**,**B**) Flow cytometry plots and enumeration for neutrophils (Gr-1^+^ and F4/80^−^) in the skin of K14cre SCFfl/fl mice and K14cre mice; (**C**,**D**) CXCL1 and CXCL2 protein levels in whole skin of mice 24 h after wounding (*p < 0.05).

During the wound closure process, the neutrophil influx is an early inflammatory response essential for the clearance of bacterial and cellular debris. Early neutrophil recruitment increases dramatically over the first 24 h after wounding^24^. Because the impairment of neutrophil recruitment is also associated with delayed wound closure^25^, we measured the neutrophil influx in the skin by flow cytometry and found that neutrophil recruitment was significantly decreased in the skin of K14cre Scf^fl/fl^ mice 24–48 h after wounding compared with K14cre control mice (Fig. 5A,B). We also assessed the number of T cells, NK cells, macrophages, and γδ T cells in the skin. However, there was no significant difference between K14cre Scf^fl/fl^ mice and K14cre control mice. To elucidate a possible cause of impaired neutrophil recruitment, we next examined CXCL1 and CXCL2 protein levels. CXCL1 levels did not change, while CXCL2 levels decreased reliably in the skin of the K14cre Scf^fl/fl^ mice 24 h after wounding as compared to the K14cre control mice (Fig. 5C,D).

### Keratinocytes express c-kit receptor to react with SCF

**Figure 6:**
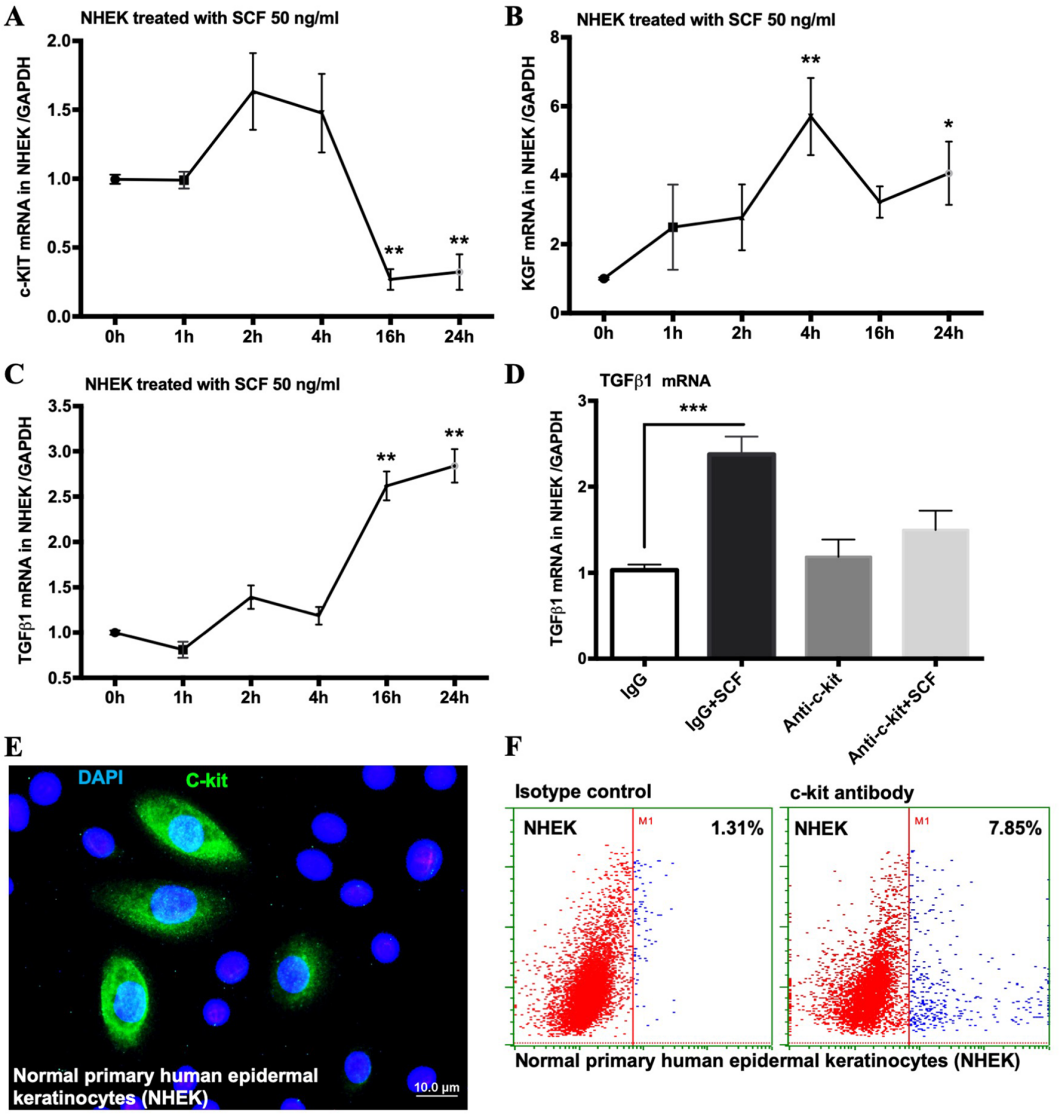
Keratinocytes express c-kit receptor to react with SCF. (**A**–**C**) qPCR analysis for c-kit, Kgf, and Tgf-β1 in NHEKs treated with 50 ng/ml SCF; (**D**) qPCR analysis for Tgf-β1 in NHEKs treated with c-kit neutralizing antibody before SCF treatment; (**E**) NHEKs were immunostained with anti-c-kit monoclonal antibody (green) and DAPI (blue); (**F**) Flow cytometry plots for c-kit expression on NHEKs (*p < 0.05, **p < 0.01, ***p < 0.001).

Studies have shown that SCF signaling is initiated through binding of SCF to the c-kit receptor resulting in subsequent auto- and paracrine activation of c-kit/JAK1/STAT3 pathway to produce growth factors such as TGF-β^11,12^. Compelling evidence has shown that SCF and its c-kit receptor are co-expressed by epidermal keratinocytes, and c-kit was found to colocalize with the epithelial cell marker desmoplakin^6,10,21^. To confirm that keratinocyte-expressed c-kit reacts with SCF, we added SCF protein to stimulate normal human primary epidermal keratinocytes (NHEKs) and measured the mRNA expressions of c-kit receptor, KGF, and TGF-β1 by real-time quantitative RT-PCR. We found that the expression of these genes changed over time after SCF treatment; e.g. c-kit mRNA expression significantly dropped at 16 and 24 h, whereas the expressions of KGF and TGF-β1 were significantly increased (Fig. 6A–C). We also observed that the increased expression of TGF-β1 was abolished when NHEKs were treated with a c-kit neutralizing antibody before SCF treatment (Fig. 6D). Furthermore, both immunofluorescence staining and flow cytometry analysis revealed that 11% ± 3.5% of NHEKs expressed c-kit receptor (Fig. 6E,F). Given that the activation of the c-kit/JAK1/STAT3 pathway by SCF to induce growth factor has already been described^11,12^, our results indicate that certain keratinocytes with the c-kit receptor can respond to SCF signal in an autocrine manner to produce growth factors for wound closure.

## Discussion

The skin is the human body’s largest organ and subserves many functions. As a result, it should come as no surprise that wound closure displays a unique and extraordinary mechanism of cascading cellular functions^26^. Although the importance of SCF in promoting wound closure has been established, the specific cellular and molecular mechanisms by which SCF improves skin wounds have remained unknown. Thus, we sought to determine whether the keratinocyte-produced SCF plays a role in skin wound closure. Our studies demonstrate that SCF production by keratinocytes is critical for wound closure without delay. We have discovered an important connection between the keratinocyte-produced SCF and skin wound closure. The mechanism identified in this paper works through the production of growth factors and chemokines in keratinocytes, which is essential for maintaining the normal wound closure process in the skin. This discovery is important as it contributes to a more complete understanding of the pathogenesis of delayed skin wound closure resulting from diabetes, old age, stress, obesity, alcoholism, smoking, or other chronic disease conditions that affect millions of people worldwide.

First, we investigated the levels of SCF in the skin of delayed wound-closure murine models, which included aged mice (14-month old), high-fat diet mice, and chronic-binge ethanol diet mice. Because separation of the epidermal sheet by dispase may have the possibility to result in imprecise estimates of gene expression, we further examined the protein levels from these same genes using Luminex and ELISA. The most impressive and important observation was a significant decrease in SCF in their epidermis and the whole skin. This finding suggests that SCF derived from keratinocytes plays a vital role in wound repair. Based on these results, we developed a mouse model that conditionally depletes SCF from the epidermal keratinocytes (K14cre Scf^fl/fl^ mice). Analysis of these mice revealed that SCF deficiency in keratinocytes resulted not only in delayed wound closure but also in significantly declined levels of the main growth factors such as KGF, TGF-β1, TGF-α and PDGF-b in the epidermis. This indicates that keratinocyte-derived SCF is critical for the production of main growth factors involved in wound closure.

Previous studies have shown that SCF signaling is initiated through the binding of SCF to the c-kit receptor resulting in subsequent auto- and paracrine activation of the c-kit/JAK1/STAT3 pathway to produce growth factors including TGF-β^11,12^. Consistent with previous evidence which showed that SCF and its c-kit receptor are co-expressed by keratinocytes^6,10,21^, our data also shows that approximately 11% of keratinocytes express c-kit receptor, which can react with SCF to initiate the production of growth factors.

It is not completely clear why only 11% of keratinocyte express c-Kit receptor. We analyzed the cell cycle of keratinocytes (data not reported) to evaluate if this expression was related to a specific cell cycle phase, but we found that expression was random in our in vitro system. More answers to this phenomenon could be found in “in vivo” two photons studies that can take into account keratinocyte position in the skin and/or in the wound^27^.

Further, we examined isolated keratinocytes from K14cre Scf^fl/fl^ mouse epidermis and found that SCF deficiency in keratinocytes not only impaired the migration of keratinocytes and normal fibroblasts but also reduced early neutrophil recruitment 24–48 h after wounding.

Reduction in neutrophil recruitment was previously described as being advantageous to wound closure. Neutrophils are extremely active in cutaneous wounds, especially in the first phase^24^. Their antimicrobial activity prevents wounds from becoming infected. However, proteases used by neutrophils to kill potential pathogens cause significant tissue damage, leading to delay in wound closure^28^. Dovi et al. also reported accelerated wound closure in neutrophil-depleted mice, which suggests that although neutrophils may provide protection against infection, they may retard wound closure^29^. However, in our system, impaired wound closure is accompanied by decreased neutrophil recruitment. We can speculate that the loss of SCF and of the growth factors controlled by SCF has a higher impact on the wound closure than the benefit generated by the decrease of neutrophil infiltration.

Epidermal regeneration is a complex process influenced by growth factors, especially the keratinocyte growth factor (KGF, also known as FGF7). Previous studies provided evidence for an important role of KGF in the repair of the injured epithelium; KGF is weakly expressed in human skin, but is strongly upregulated in dermal fibroblasts after skin injury. KGF binding to a transmembrane receptor on keratinocytes induces proliferation and migration of these cells^30^. Furthermore, KGF has been shown to protect epithelial cells from the toxic effects of reactive oxygen species. Loss of KGF, was previously described to be reduced in diabetes wound and was linked to a loss of wound contraction, that was relevant in a human model^31^ in which KGF promotes fibroblasts contraction increasing the expression of TGF beta1 and Smad pathway.

While contraction is unlikely to be functionally significant to comparable degrees across species (and particularly less so among humans), we employ a mouse model here with the expectation that the factors that govern wound-closure processes will bear key continuities with similar animals. In short, the unique and novel contribution of this work is that SCF affects other growth factors, most notably TGF-α, TGF-β1 and KGF, thus illuminating new targets in wound treatments. Taken together, the findings of this study confirmed that keratinocyte-derived SCF has a central pathogenetic role in wound closure. Our discovery is critical for developing clinical strategies to improve the body’s natural repair mechanisms and enhance treatment for patients with wound-closure pathologies.

## Methods

### Mice

C57BL/6J wild-type control mice, K14cre transgenic mice (a gift from Dr. Richard Gallo at the University of California, San Diego), Scf^fl/fl^ mice (a gift from Dr. Sean Morrison at the University of Texas Southwestern Medical Center) on a C57BL/6J background were housed at the University of California, San Diego (UCSD). All animal experiments were approved by the UCSD Institutional Animal Care and Use Committee. K14cre transgenic mice were bred with Scf^fl/fl^ mice for the generation of K14cre Scf^fl/fl^ mice. K14cre littermate controls were used in all experiments. At least three independent experiments were performed to assess reproducibility with at least four mice in each group.

### Mouse model of high-fat diet (a gift from Dr. Bernd Schnabl at UCSD)

C57BL/6 J wild-type mice (age of 6–8 weeks) were fed on a high-fat diet (Bio-Serv) for 16 weeks. Control mice received a normal diet (Bio-Serv). On a caloric basis, the high-fat diet consisted of 36% fat, 35.7% carbohydrate, and 20.5% protein (total calories: 5.49 kcal/g). The normal diet contained 5.1% fat, 65.2% carbohydrate, and 18.1% protein (total calories: 3.79 kcal/g). At the end of each trial, body weight was measured and blood glucose levels were assessed with a Roche Accu-Chek Aviva Plus blood glucose meter. These measurements were obtained following a 12 h overnight fast.

### Mouse model of chronic-binge ethanol diet (a gift from Dr. Bernd Schnabl at UCSD)

C57BL/6J wild-type mice (age of 8–10 weeks) were fed ethanol for 8 weeks (the Lieber-DeCarli diet model of chronic alcohol consumption), as previously described^32^. The Lieber-DeCarli diet comprises micro-stabilized alcohol rodent liquid diet (TestDiet), maltodextrin (TestDiet), and 200-proof ethanol (Gold Shield). The caloric intake from ethanol was 0 on day 1, 10% of total calories on days 2–4, 20% on days 5–7, 30% from day 8 until the end of 6 weeks, and 36% for the last 2 weeks. Control mice received an isocaloric amount of isomaltose instead of ethanol.

### Mouse skin wounding with punch biopsy

Mice were anesthetized with inhaled isoflurane and shaved before wounding. The dorsal skin was sterilized with 70% alcohol. Each mouse underwent a dorsal full-thickness skin wound with a 4-mm punch biopsy (Miltex, 2 biopsies/mouse). Photographs were obtained with a Canon digital camera using a ruler for each image. ImageJ software (National Institutes of Health) was used to calculate the wound area of each mouse. Wound closure analysis was evaluated by two blinded reviewers to reduce bias. The wound area was plotted as a function of time.

### Isolate epidermis from the whole skin

We isolated the epidermis from the whole skin as previously described^33^. The pieces of skin were placed dermis-side down in a Petri dish containing 10 ml of dispase solution (5 U/ml, STEMCELL Technologies). After incubation overnight at 4 °C, the pieces of skin were transferred to a new Petri dish containing calcium-free EpiLife medium (Invitrogen) to wash away any excess dispase. The epidermis was gently separated from the dermis with two pairs of curved forceps. Total RNA of the epidermis was isolated using the RNeasy Mini Kit (QIAGEN).

### Cells

Primary normal human epidermal keratinocytes (NHEKs, Life Technologies) were cultured in serum-free EpiLife medium (Invitrogen) supplemented with EpiLife Defined Growth Supplement and 60 μM CaCl_2_ (Invitrogen). For the neutralizing antibody assay, NHEKs were treated with 5 μg/ml human c-kit neutralizing antibody and normal goat IgG control (R&D Systems) for 1 h. NHEKs were then treated with 50 ng/ml SCF (R&D Systems) for 24 h.

In addition, NHEKs were stained with Alexa Fluor 488 conjugated anti-human c-kit antibody and isotype control antibody (BioLegend) for immunofluorescence images and flow cytometry analysis with the Guava EasyCyte benchtop flow cytometer (Millipore). For immunofluorescence images, slides were mounted in ProLong Anti-Fade reagent with DAPI (Molecular Probes) and imaged using a Bx51 research microscope (Olympus) and the X-Cite 120 fluorescence illumination system (EXFO Photonic Solutions). All isotype controls showed negative staining (data not shown).

### Isolate mouse dermal fibroblasts

We isolated dermal fibroblasts from mice as previously described^34^. The skin was briefly washed and minced in 2 ml phosphate-buffered saline (PBS, Invitrogen) using a syringe and an 18-gauge needle. After straining to remove large fragments, the suspension was placed in DMEM supplemented with 10% heat inactivated FBS (fetal bovine serum, Invitrogen), 100 IU/ml penicillin and 100 μg/ml streptomycin (Invitrogen). After 2 days, cells that grew from tissue fragments were transferred to 75-cm^2^ flasks and cultured to 90% confluency.

### Isolate mouse epidermal keratinocytes

We isolated the epidermal keratinocytes from mice as previously described^33^. The skin was peeled off of neonatal mice and placed dermis-side down in a Petri dish containing 10 ml of dispase solution (5 U/ml, STEMCELL Technologies). The skin was incubated at 4 °C overnight and then transferred to a new Petri dish containing Calcium-Free EpiLife medium (Invitrogen) to wash away any excess dispase. The epidermis was gently separated from the dermis with two pairs of curved forceps. 500 μl of Accutase (STEMCELL Technologies) was aliquoted into a new Petri dish. Using sterile forceps, the epidermis was slowly transferred onto the surface of the Accutase drop with the basal layer facing downward. The epidermis was incubated for 20–30 min at room temperature. 2 ml of Calcium-Free EpiLife medium was added to the epidermis. The epidermis was rubbed gently on a small area of the base of the Petri dish to separate single cells from the cell sheet. The single-cell solution was transferred to a 15 ml centrifugation tube. Rubbing was repeated after adding another 2 ml medium and the cell suspension was then collected. Cells were cultured using 12-well plates in serum-free EpiLife medium supplemented with 40 µM CaCl_2_ and EpiLife Defined Growth Supplement (Invitrogen).

### Keratinocytes scratch wound closure assay

12-well tissue culture plates were coated by 0.2 mg/ml of collagen type I solution (Sigma) for 2 h at 37 °C before rinsing with PBS. Each well was seeded with keratinocytes isolated from mouse epidermis to a final density of 200,000 cells per well and maintained at 37 °C and 5% CO_2_ for 3–4 days to permit cell adhesion and the formation of a confluent monolayer. These confluent monolayers were then scored with a sterile pipette tip to leave a scratch of approximately 0.4–0.5 mm in width. The culture medium was then immediately removed (along with any dislodged cells). The removed medium was replaced with a fresh serum-free EpiLife culture medium as described above. All scratch assays were quadruplicated.

### Fibroblasts migration assay

Fibroblasts migration towards keratinocytes was determined by using a modified Boyden chamber transwell migration assay with a membrane pore size of 8 μm as previously described^35^. Briefly, mouse epidermal keratinocytes were seeded in the lower chamber in serum-free EpiLife medium supplemented with 40 µM CaCl_2_ and EpiLife Defined Growth Supplement (Invitrogen) and maintained at 37 °C and 5% CO_2_ for 2–3 days to form an 80% confluent monolayer. 1 × 10^5^ normal C57BL/6J wild-type mouse dermal fibroblasts were treated with 10 μg/ml mitomycin C (Sigma) for 2.5 h at 37 °C with 5% CO_2_^36^, then seeded in the upper chamber in the same serum-free medium. After a 24 h incubation, non-migratory fibroblasts were carefully removed by a cotton swab from the surface of the inserts. Migratory fibroblasts that had traversed the membrane were stained with 0.1% crystal violet (Sigma). Three independent fields of migratory cells per well were photographed under microscopy and counted.

### Whole skin cell flow cytometry

Single-cell suspensions were prepared by mincing skin tissue with scissors, followed by a 60-min enzymatic digestion with 2 mg/ml collagenase Type II (Worthington), 2 mg/ml collagenase Type IV (Gibco), and 0.5 mg/ml DNase I (Roche) in PBS containing 1% bovine serum albumin (Sigma) at 37 °C under continuous stirring conditions. Digests were quenched by adding DMEM medium (Gibco) containing 10% heat-inactivated FBS and subsequently filtered through a 70-μm nylon filter (BD Biosciences). Cells were washed with DMEM before being stained with 1 μg/ml of anti-CD3, anti-NK1.1, anti-Gr-1, and anti-F4/80 monoclonal antibodies (BioLegend) according to the manufacturer’s instructions. Cells were analyzed with the Guava EasyCyte 8HT two laser, 6-color microcapillary-based benchtop flow cytometer (Millipore).

### Real-time quantitative RT-PCR

Total RNA was isolated using the RNeasy Mini Kit (QIAGEN), and 0.5 μg of total RNA was used for cDNA synthesis using the iSCRIPT cDNA Synthesis Kit (Bio-Rad). Real-time quantitative RT-PCR was performed in an ABI 7300 Real-Time PCR system (Applied Biosystems) with Taqman primers and probes (Life Technologies). We used the comparative ΔΔCT method to quantify gene expression. Target gene expression levels in the test samples were normalized to endogenous GAPDH levels and reported as fold differences relative to GAPDH gene expression in untreated baseline controls^37^. All assays were performed in triplicate and the experiments were repeated at least three times.

### Protein level measurement

Whole skin tissue weights ranging from 40 to 70 mg were homogenized in 1 ml of RIPA buffer (Sigma) with complete ULTRA Protease Inhibitor Cocktail (Roche) using bead-beating homogenizer (Precellys). An ELISA kit (R&D systems) was used to determine SCF protein levels in skin. A customized Luminex multiplex assay kit (Millipore) was used to measure KGF(FGF7), TGF-α, TGF-β1, EGF, PDGF-BB, CXCL1, and CXCL2 protein levels. All protein was detected from supernatants of tissue homogenate according to manufacturer instructions. The results were normalized to the total weight of each skin sample.

### Statistical analysis

All data are presented as the mean ± SD. At least three independent experiments were performed to assess reproducibility. Comparisons between groups were analyzed by Student's *t*-test or ANOVA for multiple comparisons. For all statistical tests, *p* values < 0.05 were considered statistically significant (*p < 0.05, **p < 0.01, ***p < 0.001).

### Study approval

All animal experimental procedures were reviewed and approved by the Institutional Animal Care and Use Committee of the University of California, San Diego (UCSD), and all experiments were performed in compliance with the institutional guidelines of UCSD.
